# The Effects of Sinapic Acid on the Development of Metabolic Disorders Induced by Estrogen Deficiency in Rats

**DOI:** 10.1155/2018/9274246

**Published:** 2018-06-04

**Authors:** Maria Zych, Ilona Kaczmarczyk-Sedlak, Weronika Wojnar, Joanna Folwarczna

**Affiliations:** ^1^Department of Pharmacognosy and Phytochemistry, School of Pharmacy with the Division of Laboratory Medicine in Sosnowiec, Medical University of Silesia, Katowice, Poland; ^2^Department of Pharmacology, School of Pharmacy with the Division of Laboratory Medicine in Sosnowiec, Medical University of Silesia, Katowice, Poland

## Abstract

Sinapic acid is a natural phenolic acid found in fruits, vegetables, and cereals, exerting numerous pharmacological effects. The aim of the study was to investigate the influence of sinapic acid on biochemical parameters related to glucose and lipid metabolism, as well as markers of antioxidant abilities and parameters of oxidative damage in the blood serum in estrogen-deficient rats. The study was performed on 3-month-old female Wistar rats, divided into 5 groups, including sham-operated control rats, ovariectomized control rats, and ovariectomized rats administered orally with estradiol (0.2 mg/kg) or sinapic acid (5 and 25 mg/kg) for 28 days. The levels of estradiol, progesterone, interleukin 18, insulin, glucose, fructosamine, lipids, and enzymatic and nonenzymatic antioxidants (superoxide dismutase, catalase, and glutathione); total antioxidant capacity; and oxidative damage parameters (thiobarbituric acid-reactive substances, protein carbonyl groups, and advanced oxidation protein products) were determined in the serum. Estradiol counteracted the carbohydrate and cholesterol metabolism disorders induced by estrogen deficiency. Sinapic acid increased the serum estradiol concentration; decreased insulin resistance and the triglyceride and total cholesterol concentrations; and favorably affected the parameters of antioxidant abilities (reduced glutathione, superoxide dismutase) and oxidative damage (advanced oxidation protein products).

## 1. Introduction

Estrogens affect the body's metabolic homeostasis by regulating many signaling pathways. Estrogens participate, among others, in the control of energy homeostasis and glucose metabolism. By acting in the hypothalamus, they control food intake, energy expenditure, and the deposition of white adipose tissue. They play an important role in the regulation of insulin secretion in the pancreas and in the response to insulin in the skeletal muscle, liver, and adipose tissue [1–3]. After menopause, estrogen deficiency contributes to the development of obesity, dyslipidemia, hypertension, and insulin resistance and, consequently, it increases the risk of cardiovascular diseases and type 2 diabetes [1, 4, 5]. The current state of knowledge on the estrogen effects on metabolism has been recently comprehensively reviewed by Mauvais-Jarvis et al. [1, 4], Sharma et al. [6], and Coyoy et al. [7].

Oxidative stress is considered to be involved in development of disorders resulting from estrogen deficiency, such as: hot flushes, cardiovascular diseases, and osteoporosis [8–10]. Numerous phenolic compounds of plant origin, which are present in food, belong to the factors that modify development of oxidative stress [11, 12]. Phenolic compounds include, among others, flavonoids and phenolic acids. The antioxidant activity of flavonoids (mainly soy isoflavones) has been the subject of studies (e.g., [13–16]) in estrogen-deficient rats. Although the antioxidant activity of phenolic acids is also well documented [17–19], there is still insufficient data on the effect of phenolic acids on oxidative stress in conditions of estrogen deficiency. Soy isoflavones are phytoestrogens, and their effect on oxidative stress parameters may result from their effects on estrogen receptors. Phenolic acids have rather not been demonstrated to have affinity for estrogen receptors [20]; however, our earlier studies indicated that some hydroxycinnamic phenolic acids (especially caffeic acid) may increase the serum estradiol levels in estrogen-deficient rats [21].

Sinapic acid is a phenolic acid, found, among others, in fruits (e.g., strawberries or lemons), grains (oat), and vegetables (especially from the Brassicaceae family, like tronchuda cabbage, broccoli, and turnip) as well as in some medicinal plants and species (for instance borage, sage, mace, or rosemary) [22–24]. Sinapic acid, a hydroxycinnamic acid derivative, has antioxidant activity. There are reports on its numerous peripheral activities (anti-inflammatory [25], hypoglycemic [26, 27], cardioprotective [28, 29], hepatoprotective [30], and nephroprotective [31]) and central activities (neuroprotective [32–34], anticonvulsant [34], and anxiolytic [35]).

Taking into account that phenolic acids may influence both the oxidative stress parameters and estrogen levels, the aim of the study was to investigate the effects of administration of sinapic acid on the serum biochemical parameters related to the development of metabolic disorders resulting from estrogen deficiency in rats. So far, there are no data on the effects of sinapic acid on parameters related to lipid and glucose metabolism in conditions of estrogen deficiency.

## 2. Materials and Methods

### 2.1. Animals and Drugs

The experiment was conducted on mature, 3-month-old female Wistar rats, with the approval of the Local Ethics Commission in Katowice (permission numbers 38/2015, 148/2015, and 66/2016). The animals were provided by the Center of Experimental Medicine, Medical University of Silesia, Katowice, Poland. Rats had unlimited access to drinking water and standard laboratory feed (Labofeed B; Wytwórnia Pasz “Morawski”, Kcynia, Poland). The following substances and drugs were used: sinapic acid (Sigma-Aldrich, St. Louis, MO, USA), estradiol hemihydrate (Estrofem, Novo Nordisk A/S, Bagsvard, Denmark), ketamine (Ketamina 10%, Biowet Puławy, Puławy, Poland), xylazine (Xylapan, Vetoquinol Biowet, Gorzów Wlkp., Poland).

### 2.2. Experimental Design

During the 13-day adaptation period, the animals were divided into the following groups (*n* = 10): SHAM—sham-operated control rats; OVX—ovariectomized control rats; ESTR—ovariectomized rats receiving estradiol (0.2 mg/kg p.o. daily); SA5—ovariectomized rats receiving sinapic acid (5 mg/kg p.o. daily); SA25—ovariectomized rats receiving sinapic acid (25 mg/kg p.o. daily). The rats of the ESTR group served as a positive control group. Bilateral ovariectomy (in rats of the OVX, ESTR, SA5, and SA25 groups) and sham surgery (in rats of the SHAM group) were performed 7 days before the start of the estradiol or sinapic acid administration. The animals were anesthetized with the mixture of ketamine and xylazine (87.5 and 12.5 mg/kg i.p., resp.). Sinapic acid and estradiol were administered orally by an intragastric tube once a day for 4 weeks as water suspension prepared with the addition of Tween 20 q.s. (maximum 1 *μ*l of Tween 20 per 1 ml of water). The doses were within the range of doses administered in previous experimental studies of sinapic acid [27, 28, 30, 31, 33] and estradiol [36] in rats. The SHAM and OVX control rats received water with the same amount of Tween 20, in the same volume of 2 ml/kg p.o. All animals were weighed twice a week. The final measurements of the body mass were made before the last administration of sinapic acid, estradiol, or vehicle.

The day after the last administration of sinapic acid or estradiol, the rats were sacrificed by cardiac exsanguination under ketamine/xylazine anesthesia. The animals were fasted overnight prior to euthanasia. The serum was obtained from the clotted blood by centrifugation and frozen until the biochemical measurements were performed. The uterus, thymus, liver, and right kidney were isolated from the sacrificed rats and weighed. All spectrophotometric measurements were carried out using the Tecan Infinite M200 PRO plate reader with Magellan 2.0 software.

**Figure 1:**
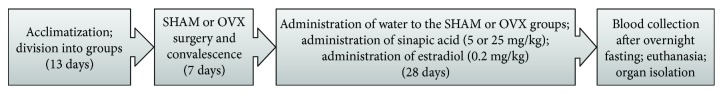
Timeline of the experiment.

The timeline of the experiment is shown in Figure 1.

### 2.3. Determination of Sex Hormones

The serum concentrations of estradiol and progesterone were determined by ELISA, using DiaMetra (Segrate-Milano, Italy) kits according to instructions provided by the manufacturer.

### 2.4. Determination of Parameters Related to Glucose Homeostasis

The serum concentrations of glucose and fructosamine were determined spectrophotometrically, using Pointe Scientific (Canton, MI, USA) kits, while insulin concentration was determined by ELISA, using a BioVendor (Brno, Czech Republic) kit, according to the manufacturer's instructions. The HOMA-IR index (homeostasis model assessment for insulin resistance) was calculated using formula (1) [37, 38]:

(1) $$\begin{array}{c} HOMA‐IR=\frac{\left[fasting\;glucose\;\left(mg/dl\right)\times fasting\;insulin\;\left(\mu U/ml\right)\right]}{405}. \end{array}$$

### 2.5. Determination of Lipid Concentrations

The serum concentrations of triglycerides, total cholesterol, low-density lipoprotein cholesterol (LDL-C), and high-density lipoprotein cholesterol (HDL-C) were determined spectrophotometrically using Pointe Scientific (Canton, MI, USA) kits, according to instructions provided by the manufacturer.

### 2.6. Determination of Nonenzymatic and Enzymatic Antioxidants

The serum reduced glutathione (GSH), and oxidized glutathione (GSSG) levels, total antioxidant capacity (TAC), superoxide dismutase (SOD) activity, and catalase (CAT) activity were determined using Cayman Chemical (Ann Arbor, MI, USA) kits.

The activities of SOD and CAT were converted to mg of protein. The serum protein level was determined by the biuret method with the use of a Pointe Scientific (Canton, MI, USA) kit. The measurements were performed according to the instructions provided by the manufacturers.

### 2.7. Determination of Oxidation Damage Parameters

The serum content of TBARS (thiobarbituric acid-reactive substances) was determined using the method of Ohkawa et al. [39]. The method is based on the reaction between lipid peroxidation products and thiobarbituric acid. The intensity of the resulting color was determined spectrophotometrically at the wavelength of 535 nm. To establish a standard curve, 1,1,3,3-tetraethoxypropane (Sigma-Aldrich, St. Louis, MO, USA) was used.

Spectrophotometric determination of advanced oxidation protein products (AOPP) was carried out on the basis of the protocol described by Witko-Sarsat et al. [40]. The calibration curve was made using chloramine T (Sigma-Aldrich, St. Louis, MO, USA), and the absorbance was measured at the wavelength of 340 nm. The concentration of AOPP was presented in *μ*mol of chloramine T equivalents/l.

The concentration of protein carbonyl groups (PCG) was measured spectrophotometrically using a commercially available kit (Cell Biolabs, San Diego, CA, USA), according to the instructions provided by the manufacturer.

### 2.8. Determination of Interleukin 18

The serum concentration of interleukin 18 (IL-18) was determined by ELISA with the use of a Cloud-Clone (Houston, TX, USA) kit, following the instructions provided by the manufacturer.

### 2.9. Determination of Biochemical Markers of Liver and Kidney Function

The activities of aspartate aminotransferase (AST) and alanine aminotransferase (ALT), as well as the concentrations of uric acid and urea, were determined spectrophotometrically with the use of kits produced by BioSystems (Costa Brava, Barcelona, Spain) in the serum. The concentration of creatinine was determined using a Pointe Scientific (Canton, MI, USA) kit. The measurements were made according to the instructions provided by manufacturers.

### 2.10. Statistical Analysis

The results are presented as the arithmetic mean ± SEM. To assess the statistical significance of the results, one-way ANOVA followed by Fisher's LSD post hoc test was used. The results were considered statistically significant with *p* ≤ 0.05.

## 3. Results

### 3.1. Effect of Estradiol and Sinapic Acid on the Body Mass and Organ Mass

The body mass and body mass gain after 4 weeks of observation increased statistically significantly in the ovariectomized control rats (OVX) compared to the sham-operated control rats (SHAM). In the OVX control rats, the mass of the uterus decreased significantly, while the mass of the thymus increased. The mass of the liver and kidney did not change significantly in comparison with the mass of these organs in the SHAM control rats. Administration of estradiol at a dose of 0.2 mg/kg caused a decrease in the body mass gain, an increase in the uterine mass, and a reduction in the thymus mass, while it did not affect the liver and kidney mass, as compared to the OVX control rats. Sinapic acid administration at both doses did not lead to any changes in the body mass, body mass gain, and mass of all examined organs (Table 1).

### 3.2. Effect of Estradiol and Sinapic Acid on the Concentration of Sex Hormones in the Serum

**Figure 2:**
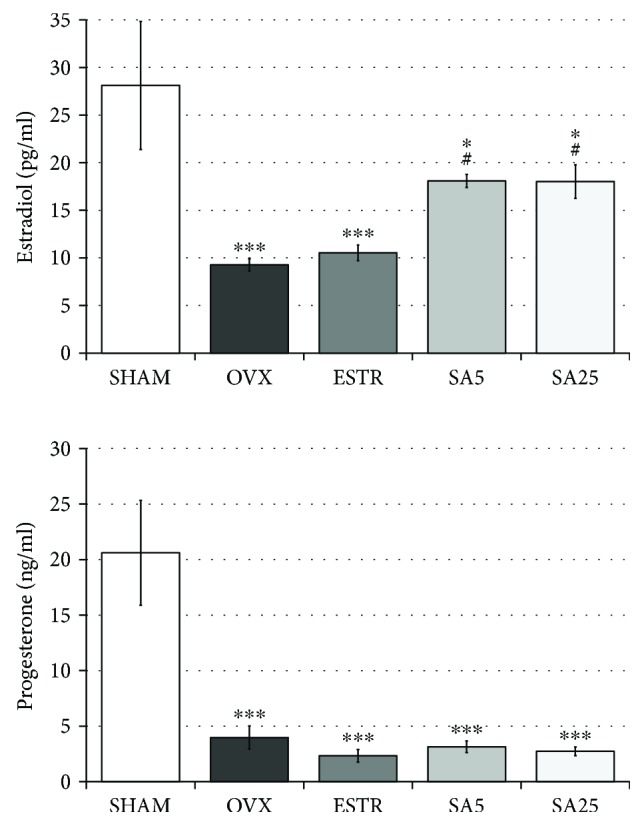
Effect of estradiol and sinapic acid on the serum concentration of estradiol and progesterone in ovariectomized rats. Sinapic acid at doses of 5 mg/kg (SA5) and 25 mg/kg (SA25) or estradiol ((ESTR) 0.2 mg/kg) was administered orally to ovariectomized rats, once daily for 28 days. SHAM: sham-operated control rats; OVX: ovariectomized control rats. Results are presented as the mean ± SEM. One-way ANOVA followed by Fisher's LSD test was used for evaluation of statistical significance of the results. ^∗^*p* ≤ 0.05, ^∗∗∗^*p* < 0.001: significantly different from the SHAM control rats. ^#^*p* ≤ 0.05: significantly different from the OVX control rats.

In the OVX control rats, a statistically significant reduction in the estradiol and progesterone concentrations was observed, compared to the SHAM control rats. Administration of estradiol did not trigger any changes in the levels of both hormones tested. Sinapic acid administration at both used doses resulted in a statistically significant increase in the serum estradiol concentration in comparison with the OVX control rats. Sinapic acid did not affect the serum progesterone concentration (Figure 2).

### 3.3. Effect of Estradiol and Sinapic Acid on the Glucose Homeostasis Parameters in the Serum

**Figure 3:**
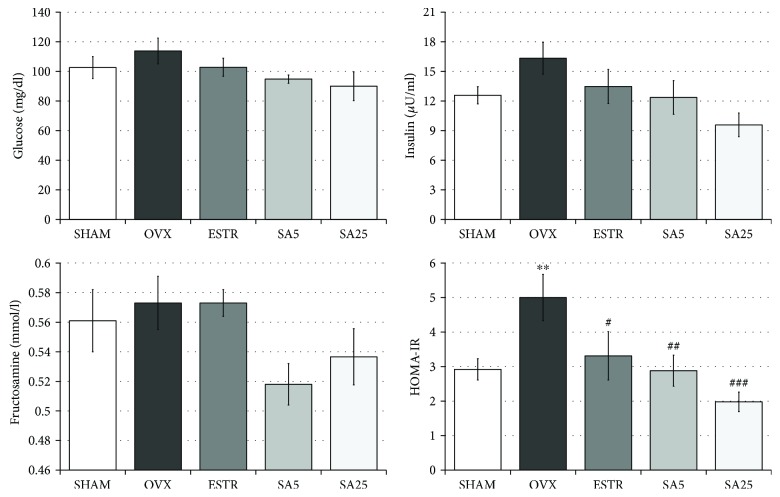
Effect of estradiol and sinapic acid on the serum parameters related to glucose homeostasis in ovariectomized rats. HOMA-IR: homeostasis model assessment of insulin resistance. Sinapic acid at doses of 5 mg/kg (SA5) and 25 mg/kg (SA25) or estradiol ((ESTR) 0.2 mg/kg) was administered orally to ovariectomized rats, once daily for 28 days. SHAM: sham-operated control rats; OVX: ovariectomized control rats. Results are presented as the mean ± SEM. One-way ANOVA followed by Fisher's LSD test was used for evaluation of statistical significance of the results. ^∗∗^*p* < 0.01: significantly different from the SHAM control rats. ^#^*p* ≤ 0.05, ^##^*p* < 0.01, and ^###^*p* < 0.001: significantly different from the OVX control rats. No statistically significant differences in results for glucose, insulin, and fructosamine were demonstrated in ANOVA.

In the OVX control rats, no statistically significant changes in the glucose, insulin, and fructosamine concentrations were found; however, the HOMA-IR index significantly increased compared to that of the SHAM control rats. Estradiol and sinapic acid at both doses did not significantly affect the glucose, insulin, and fructosamine concentrations, but they led to a reduction in the HOMA-IR index, compared to that of the OVX control rats. Sinapic acid at a dose of 5 mg/kg showed a tendency to lower fructosamine concentration, while the higher dose (25 mg/kg) revealed a tendency to lower the insulin concentration in the serum of OVX rats (Figure 3).

### 3.4. Effect of Estradiol and Sinapic Acid on the Concentration of Lipids in the Serum

**Figure 4:**
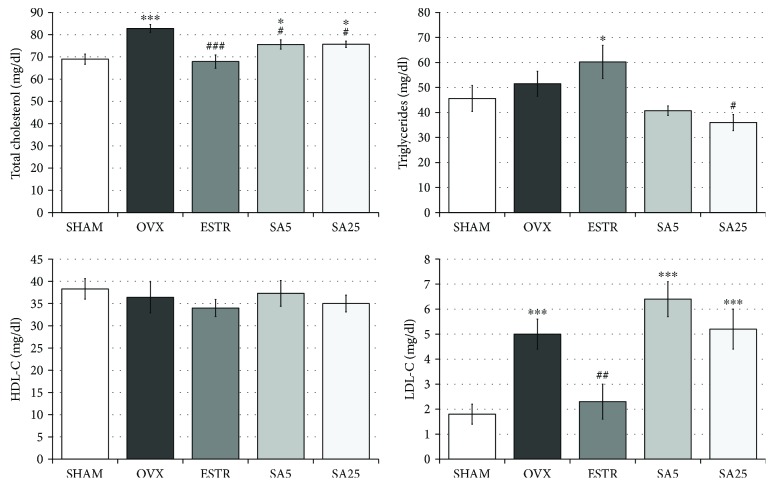
Effect of estradiol and sinapic acid on the serum lipid levels in ovariectomized rats: total cholesterol, triglycerides, high-density lipoprotein cholesterol (HDL-C), and low-density lipoprotein cholesterol (LDL-C). Sinapic acid at doses of 5 mg/kg (SA5) and 25 mg/kg (SA25) or estradiol ((ESTR) 0.2 mg/kg) was administered orally to ovariectomized rats, once daily for 28 days. SHAM: sham-operated control rats; OVX: ovariectomized control rats. Results are presented as the mean ± SEM. One-way ANOVA followed by Fisher's LSD test was used for evaluation of statistical significance of the results. ^∗^*p* ≤ 0.05, ^∗∗∗^*p* < 0.001: significantly different from the SHAM control rats. ^#^*p* ≤ 0.05, ^##^*p* < 0.01, and ^###^*p* < 0.001: significantly different from the OVX control rats. No statistically significant differences in results for HDL-C were demonstrated in ANOVA.

In the OVX control rats, a statistically significant increase in the total cholesterol and LDL-C concentrations was found, while no changes in the triglyceride and HDL-C concentrations were observed in comparison to those in the SHAM control rats. Administration of estradiol caused decreases in the total cholesterol and LDL-C concentrations as compared to the OVX control rats, without any significant effect on the triglyceride and HDL-C concentrations. Sinapic acid at both doses lowered the total cholesterol concentration and, when used at a dose of 25 mg/kg, it decreased the triglyceride concentration. No effect of sinapic acid on the HDL-C and LDL-C concentrations was observed (Figure 4).

### 3.5. Effect of Estradiol and Sinapic Acid on the Concentration of Nonenzymatic and Enzymatic Antioxidants in the Serum

In the group of the OVX control rats, a significant decrease in the GSH concentration, tendency to decrease the GSH/GSSG ratio, and nonsignificant decrease in TAC were found as compared to the SHAM control rats. There was no effect on the GSSG concentration. The SOD activity was significantly increased; an increase in the CAT activity was not significant. Administration of estradiol resulted in the increased GSH concentration and decreased SOD activity. Administration of sinapic acid at the lower dose resulted in a tendency to increase the GSH concentration, and its administration at the higher dose led to a statistically significant increase in this parameter in comparison to the OVX control rats. Only the higher sinapic acid dose reduced the activity of SOD. Other parameters did not change significantly after administration of estradiol or sinapic acid (Table 2).

### 3.6. Effect of Estradiol and Sinapic Acid on the Concentration of Oxidative Damage Indicators in the Serum

**Figure 5:**
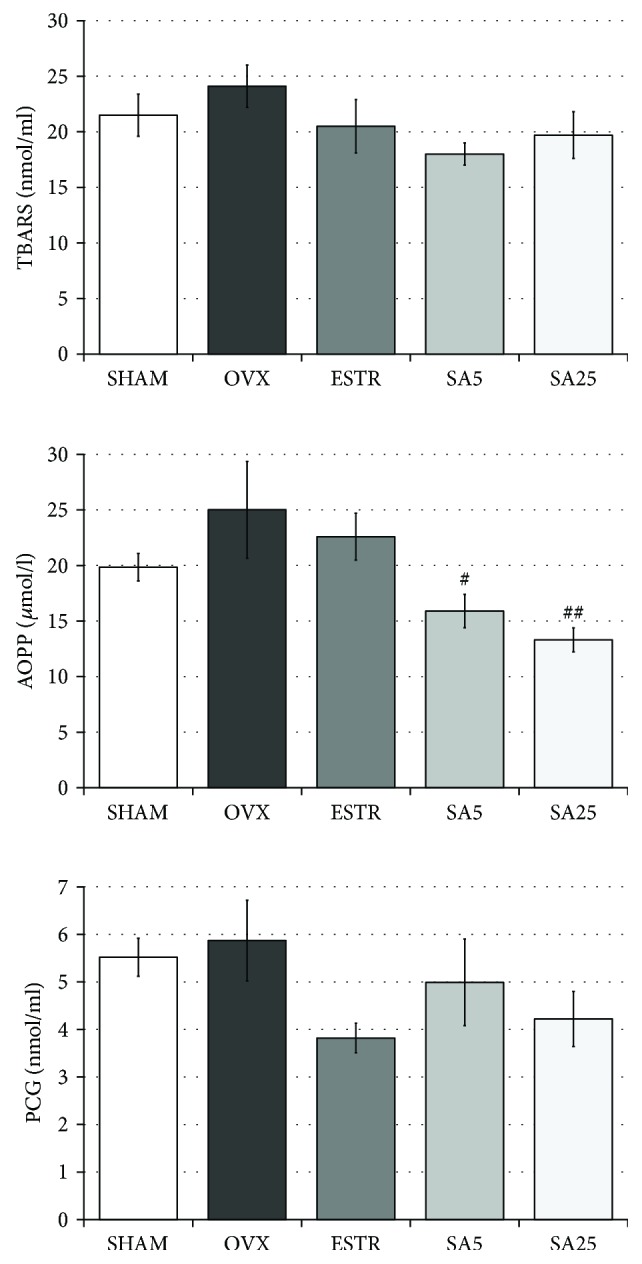
Effect of estradiol and sinapic acid on the serum concentrations of oxidative damage markers: thiobarbituric acid-reactive substances (TBARS), advanced oxidation protein products (AOPP), and protein carbonyl groups (PCG) in ovariectomized rats. Sinapic acid at doses of 5 mg/kg (SA5) and 25 mg/kg (SA25) or estradiol ((ESTR) 0.2 mg/kg) was administered orally to ovariectomized rats, once daily for 28 days. SHAM: sham-operated control rats; OVX: ovariectomized control rats. Results are presented as the mean ± SEM. The concentration of AOPP is presented in chloramine T equivalents. One-way ANOVA followed by Fisher's LSD test was used for evaluation of statistical significance of the results. ^#^*p* ≤ 0.05, ^##^*p* < 0.01: significantly different from the OVX control rats. No statistically significant differences in results for TBARS and PCG were demonstrated in ANOVA.

Estrogen deficiency in the OVX control rats resulted in insignificant increases in the concentrations of TBARS, AOPP, and PCG compared to those in the SHAM control rats. Administration of estradiol did not significantly affect the level of the oxidative damage parameters investigated, although there was a tendency to decrease the PCG concentration in comparison with the OVX control rats. Sinapic acid at both doses significantly lowered the AOPP concentration in the OVX rats but did not cause any significant changes in the concentration of PCG. Sinapic acid at both doses showed a tendency to lower the concentration of TBARS in the OVX rats (Figure 5).

### 3.7. Effect of Estradiol and Sinapic Acid on the IL-18 Concentration in the Serum

**Figure 6:**
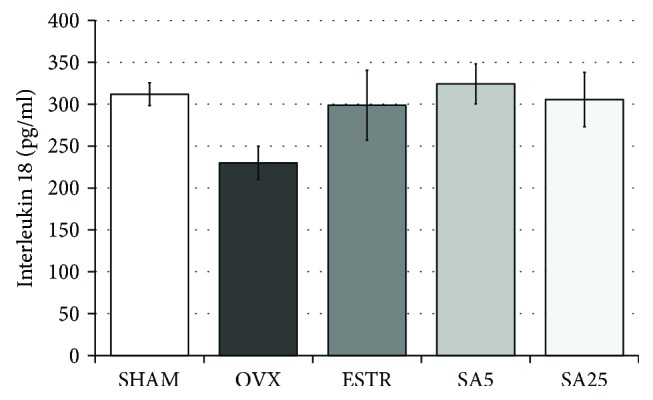
Effect of estradiol and sinapic acid on the serum concentration of interleukin 18 (IL-18) in ovariectomized rats. Sinapic acid at doses of 5 mg/kg (SA5) and 25 mg/kg (SA25) or estradiol ((ESTR) 0.2 mg/kg) was administered orally to rats, once daily for 28 days. SHAM: sham-operated control rats; OVX: ovariectomized control rats. Results are presented as the mean ± SEM. One-way ANOVA was used for evaluation of statistical significance of the results. No statistically significant differences were demonstrated in ANOVA.

In the OVX control rats, a tendency to decrease the IL-18 concentration, compared to the SHAM control rats, was observed. Administration of estradiol and sinapic acid at both doses resulted in a statistically insignificant increase in the IL-18 concentration in comparison with the OVX control rats (Figure 6).

### 3.8. Effect of Estradiol and Sinapic Acid on the Serum Biochemical Markers of Liver and Kidney Function

Estrogen deficiency did not alter the AST or ALT activity as well as the concentration of uric acid, urea, and creatinine in comparison with that of the SHAM control rats. Administration of estradiol or sinapic acid at both doses did not affect these parameters, when compared to the OVX control rats (Table 3).

## 4. Discussion

In the present study, attempting to investigate the effect of sinapic acid on biochemical parameters related to glucose and lipid metabolism as well as oxidative stress in the blood serum in conditions of estrogen deficiency, the rats were administered two doses of sinapic acid: 5 and 25 mg/kg p.o. The content of sinapic acid in food products is reported to be very diverse. For instance, it varies from 0.26 *μ*g/g to 450.3 *μ*g/g in fruits, from 0.5 *μ*g/g to 180.1 *μ*g/g in vegetables, and from 0.07 to 56.05 *μ*g/g in cereals [22]. Assuming a daily intake of 150–250 g of vegetables or fruits rich in sinapic acid by an adult human weighing 70 kg and taking into consideration much faster metabolism in rats [41], it can be assumed that the dose of 5 mg/kg corresponds to maximum human doses achievable by a high dietary intake. The higher dose used in this study (25 mg/kg) was more effective in lowering glucose than the 5 mg/kg dose in rats with experimental type 1 diabetes [27].

In the present study, 5 weeks after performing the bilateral ovariectomy, decreases in the serum levels of sex hormones (estradiol and progesterone) and characteristic changes in estrogen-dependent organ mass (a decreased uterine mass and increased thymus mass), as well as an increased body mass gain, were demonstrated, indicating that the rats were estrogen deficient. Typical disorders of carbohydrate metabolism (insulin resistance) and lipid homeostasis (increases in the total cholesterol and LDL-C concentrations) were observed, consistent with literature data [21, 42, 43].

Although 24 h after the last estradiol administration its serum level did not differ from that of the control rats (due to the fact that the free estradiol half-life in rat circulation is only 2 hours [44]), the estrogenic effect was manifested by an increase in the uterus mass and a decrease in the thymus mass. Administration of estradiol at a dose 0.2 mg/kg p.o. for 4 weeks had a counteracting effect on all the abovementioned disturbances of carbohydrate and lipid metabolism resulting from estrogen deficiency, consistent with the literature data [45, 46].

After administration of sinapic acid at both doses, there was an increase in the serum estradiol concentration, without effect on the progesterone concentration. The increase in the estradiol concentration is consistent with the results of our previous study [21], in which we demonstrated that another phenolic acid (caffeic acid; 10 mg/kg p.o. daily for 28 days) increased the estradiol level in the serum of estrogen-deficient rats. This suggests the possibility of enhancing the estradiol synthesis in extraovarian tissues (muscle, adipose tissue) by different phenolic acids. Both caffeic acid and sinapic acid are derivatives of hydroxycinnamic acid: *3,4-dihydroxycinnamic acid* (caffeic acid) and *3,5-dimethoxy-4-hydroxycinnamic acid* (sinapic acid). Interestingly, administration of sinapic acid did not lead to any estrogen-specific changes in the estrogen-dependent organ mass (such as the increased uterine mass or decreased thymus mass). The uterine mass in ovariectomized rats receiving sinapic acid even showed a strong tendency to decrease, which in turn may indicate antiestrogenic activity in the uterus.

In our study, sinapic acid reduced insulin resistance determined by the calculated HOMA-IR index, which is widely used to assess insulin sensitivity in humans and experimental animals [8, 36, 37]. Reduction of this parameter both by estradiol and sinapic acid may indicate that sinapic acid acts via estrogen pathways. The beneficial effect of sinapic acid on glucose homeostasis was previously demonstrated in rat models of type 1 diabetes induced by streptozotocin and type 2 diabetes induced by a fructose-rich diet [27]. In type 1 diabetes, sinapic acid reduced the glucose concentration in plasma and increased GLUT4 gene expression in skeletal muscle [27]. In type 2 diabetes, sinapic acid increased the sensitivity of cells to insulin [27].

In this study, administration of sinapic acid at both doses reduced the total cholesterol concentration and, at the higher dose, it also lowered the triglyceride concentration in ovariectomized rats. The results concerning the lowering of the level of total cholesterol are consistent with our previous study on other phenolic acids (caffeic, p-coumaric, and chlorogenic acids) [21]. Also the beneficial effect of sinapic acid on the lipid profile has been demonstrated in the isoproterenol-induced myocardial infarction model [28] and L-NAME-induced hypertension [29]. In those models, sinapic acid reduced the activity of HMG-CoA reductase (the rate-controlling enzyme in the cholesterol synthesis pathway) in the liver [28, 29] and serum [29]. However, in the present study, no effect of sinapic acid on the LDL-C serum concentration was observed, as opposed to estradiol.

In order to broaden the knowledge about mechanisms of action of sinapic acid on the development of metabolic disorders induced by estrogen deficiency, the studies on oxidative stress parameters in the serum were carried out. It is suggested that estradiol is protective against oxidative damage prior to menopause [9]. Numerous *in vivo* studies performed in the rat model of menopause induced by bilateral ovariectomy have shown that, under estrogen deficiency, the oxidative-antioxidative balance is disturbed, which manifests in the changes in antioxidative parameters, both enzymatic and nonenzymatic, as well as in oxidative damage parameters in various organs [42, 47–54]. However, the number of reports on the measurement of those parameters in the serum of ovariectomized experimental animals is less numerous [42, 54–58].

The most commonly studied enzyme markers of antioxidative abilities are superoxide dismutase (SOD), catalase (CAT), and glutathione peroxidase (GPx). These enzymes form an integrated, very sensitive, and specific antioxidant system [59]. SOD participates in the neutralization of superoxide anion radicals, while CAT and GPx break down the hydrogen peroxide formed as a result of SOD activity. In our study, in the OVX control rats, there were increases in the SOD and CAT activities, consistent with the results of previous studies [55]. However, it should be pointed out that there are also reports indicating the reduction of activity of these enzymes in the serum of ovariectomized rats [56]. GSH (reduced glutathione), an important nonenzymatic antioxidant, is a major nonproteinaceous thiol, which protects proteins against oxidation [60]. In our study, the concentration of GSH in the serum of estrogen-deficient rats decreased, which is consistent with previous observations [55, 57]. Moreover, the serum level of TAC, statistically insignificantly decreased. Similar results in ovariectomized rats have been reported earlier [58].

The oxidative damage parameters of lipids (TBARS) and protein (PCG, AOPP) in the serum were also evaluated. Under the influence of estrogen deficiency, in animal models, the concentration of TBARS usually increases [42, 43, 54], although there are also publications indicating the lack of changes in TBARS [55]. The concentration of PCG also increases in estrogen deficiency [42]; to our knowledge, there are no reports describing the effect of estrogen deficiency on the AOPP concentration in laboratory animals. In our experiment, 5 weeks after ovariectomy, the concentrations of all oxidative damage indicators examined (TBARS, PCG, and AOPP) insignificantly increased.

In the present study, the effects of sinapic acid and estradiol on some antioxidant parameters were similar. Under the influence of sinapic acid and estradiol, the concentration of GSH in the serum increased, and, in case of estradiol and the higher dose of sinapic acid, the activity of SOD decreased. Those particular activities of sinapic acid may partly result from an increase in the serum estradiol levels.

On the other hand, sinapic acid, unlike estradiol, improved one of the oxidative damage parameters—AOPP. Apart from being markers of oxidative damage, AOPP are recognized as markers of inflammation [40, 61]. AOPP bind to RAGE receptor, which is the receptor for advanced glycation products (AGEs). Stimulation of this receptor results in production of proinflammatory cytokines and adhesion molecules, which exacerbates inflammation and activates NADPH oxidase, which in turn induces ROS formation and intensification of oxidative stress [62]. The decrease of the AOPP concentration in the serum of ovariectomized rats under the influence of sinapic acid indicates the potential of sinapic acid to reduce oxidative stress and exert anti-inflammatory activity. Another manifestation of antioxidative properties of sinapic acid is a tendency to decrease the TBARS concentration in the serum of ovariectomized rats. The antioxidant activity of sinapic acid may be due to the fact that the hydroxycinnamic acid derivatives neutralize free radicals by liberating a hydrogen atom followed by the formation of a phenoxy radical. The resulting radical is stabilized by the conjugated system of the arene and the alkenyl carboxylate side chain [23, 24].

In addition to the oxidative stress parameters, the concentration of a proinflammatory cytokine IL-18 was examined. IL-18 was included in the research because its concentration was reported to be estradiol dependent [63]. However, the role of IL-18 in metabolic syndrome, diabetes, or obesity is not clear [64–68]. In our study, in the ovariectomized rats, which were characterized by increased body mass and insulin resistance, a reduced concentration of IL-18 was demonstrated and administration of estradiol caused an insignificant increase in the IL-18 concentration, while administration of sinapic acid (5 mg/kg) resulted in a strong tendency to increase the level of IL-18 in relation to the ovariectomized controls. Similarly, Russell et al. [63] reported a slight increase in IL-18 levels in estradiol-treated ovariectomized rats fed a standard diet. However, an estradiol-induced decrease in IL-18 concentration was demonstrated in ovariectomized rats fed an isoflavone-free diet [63]. The present study was performed in rats fed a standard laboratory diet containing soy. Taking into consideration the abovementioned results of Russell et al. [63], it is possible that the effects of sinapic acid on the IL-18 concentration and other parameters observed in the present study also may depend on the diet. Such a possibility should be taken into account because, in case of caffeic acid, the estradiol increasing effect depended on the diet [21, 36]. Although administration of caffeic acid (10 mg/kg p.o.) in ovariectomized rats fed standard laboratory diet resulted in an increase in the serum estradiol concentration [21], the administration of caffeic acid (5 and 50 mg/kg p.o.) did not increase the estradiol concentration in rats fed a soy-free diet (and with low content of phenolic acids) [36]. Results of the present study suggest that more data on the impact of food phytoestrogens on the activity of other diet components or medicines are needed.

## 5. Conclusion

In conclusion, administration of sinapic acid to ovariectomized rats reduced the concentration of total cholesterol, triglyceride, and HOMA-IR index, and also normalized some serum parameters of antioxidative abilities and oxidative damage, which were disordered by estrogen deficiency. The effects of sinapic acid could partly be due to an increase in the serum estradiol concentration. However, differential effects of sinapic acid and estradiol (e.g., on the body mass gain and uterine mass, the serum concentrations of triglycerides, LDL-C, and AOPP) indicate that sinapic acid may also act according to mechanisms other than estrogenic.

## Figures and Tables

**Table 1:** Effect of estradiol and sinapic acid on the body mass gain and mass of selected organs in ovariectomized rats.

Parameter/group	SHAM	OVX	ESTR	SA5	SA25
Body mass at the start of drug administration (g)	212.2 ± 3.0	222.0 ± 4.3	222.5 ± 3.0	225.0 ± 4.8	224.4 ± 4.9
Body mass after 4 weeks of drug administration (g)	235.3 ± 3.5	271.8 ± 5.0^∗∗∗^	257.7 ± 4.3^∗∗^	272.1 ± 6.5^∗∗∗^	271.0 ± 6.4^∗∗∗^
Body mass gain after 4 weeks (g)	23.1 ± 1.9	49.8 ± 1.8^∗∗∗^	35.2 ± 3.2^∗∗^^###^	47.1 ± 2.4^∗∗∗^	46.6 ± 2.7^∗∗∗^
Uterus mass (g)	0.434 ± 0.036	0.082 ± 0.005^∗∗∗^	0.181 ± 0.010^∗∗∗^^###^	0.068 ± 0.005^∗∗∗^	0.071 ± 0.002^∗∗∗^
Thymus mass (g)	0.377 ± 0.022	0.596 ± 0.044^∗∗∗^	0.500 ± 0.023^∗^^#^	0.625 ± 0.037^∗∗∗^	0.613 ± 0.038^∗∗∗^
Kidney mass (g)	0.758 ± 0.020	0.789 ± 0.016	0.785 ± 0.029	0.760 ± 0.014	0.768 ± 0.013
Liver mass (g)	5.906 ± 0.149	6.382 ± 0.156	6.518 ± 0.132	6.365 ± 0.315	6.330 ± 0.175

Sinapic acid at doses of 5 mg/kg (SA5) and 25 mg/kg (SA25) or estradiol ((ESTR) 0.2 mg/kg) was administered orally to rats, once daily for 28 days. SHAM: sham-operated control rats; OVX: ovariectomized control rats. Results are presented as the mean ± SEM. One-way ANOVA followed by Fisher's LSD test was used for evaluation of statistical significance of the results.^∗^*p* ≤ 0.05, ^∗∗^*p* < 0.01, and ^∗∗∗^*p* < 0.001: significantly different from the SHAM control rats. ^#^*p* ≤ 0.05, ^###^*p* < 0.001: significantly different from the OVX control rats. No statistically significant differences in results for the body mass at the start of drug administration, as well as kidney and liver mass, were demonstrated in ANOVA.

**Table 2:** Effect of estradiol and sinapic acid on the serum concentrations of nonenzymatic antioxidants and the serum activity of antioxidative enzymes.

Parameter/group	SHAM	OVX	ESTR	SA5	SA25
GSH (nmol/ml)	1.197 ± 0.032	1.090 ± 0.024^∗^	1.187 ± 0.041^#^	1.183 ± 0.033	1.239 ± 0.026^##^
GSSG (nmol/ml)	0.305 ± 0.013	0.357 ± 0.030	0.317 ± 0.027	0.377 ± 0.024	0.324 ± 0.029
GSH/GSSG	3.994 ± 0.245	3.161 ± 0.266	3.892 ± 0.356	3.231 ± 0.297	4.060 ± 0.532
TAC (*μ*mol/ml)	1.27 ± 0.18	0.89 ± 0.06	1.07 ± 0.13	1.18 ± 0.21	0.94 ± 0.04
SOD (U/mg of protein)	5.22 ± 0.19	6.20 ± 0.34^∗∗^	5.35 ± 0.15^##^	5.87 ± 0.14^∗^	5.57 ± 0.16^#^
CAT (nmol/min/mg of protein)	0.52 ± 0.08	0.74 ± 0.19	0.60 ± 0.08	0.70 ± 0.13	0.63 ± 0.10

Sinapic acid at doses of 5 mg/kg (SA5) and 25 mg/kg (SA25) or estradiol ((ESTR) 0.2 mg/kg) was administered orally to rats once daily for 28 days. SHAM: sham-operated control rats; OVX: ovariectomized control rats; GSH: reduced glutathione; GSSG: oxidized glutathione; TAC: total antioxidant capacity; SOD: superoxide dismutase; CAT: catalase. Results are presented as the mean ± SEM. The level of TAC is presented in Trolox equivalents. One unit of SOD was the amount of enzyme needed to exhibit 50% dismutation of the superoxide radical. One-way ANOVA followed by Fisher's LSD test was used for evaluation of statistical significance of the results. ^∗^*p* ≤ 0.05, ^∗∗^*p* < 0.01: significantly different from the SHAM control rats. ^#^ *p* ≤ 0.05, ^##^*p* < 0.01: significantly different from the OVX control rats. No statistically significant differences in results for GSSG, GSH/GSSG ratio, TAC, and CAT were demonstrated in ANOVA.

**Table 3:** Effect of estradiol and sinapic acid on the serum biochemical markers of liver and kidney function in ovariectomized rats.

Parameter/group	SHAM	OVX	ESTR	SA5	SA25
AST (U/l)	39.22 ± 3.38	34.72 ± 2.78	35.83 ± 3.62	38.14 ± 4.08	40.35 ± 3.73
ALT (U/l)	23.68 ± 2.19	23.52 ± 1.38	22.78 ± 1.83	23.69 ± 2.08	28.38 ± 3.61
Uric acid (mg/dl)	1.13 ± 0.07	1.18 ± 0.17	1.10 ± 0.10	0.86 ± 0.06	1.00 ± 0.06
Urea (mg/dl)	25.72 ± 4.74	35.36 ± 4.19	38.45 ± 5.61	32.46 ± 3.63	37.49 ± 2.46
Creatinine (mg/dl)	0.449 ± 0.022	0.476 ± 0.019	0.431 ± 0.035	0.472 ± 0.031	0.415 ± 0.053

Sinapic acid at doses of 5 mg/kg (SA5) and 25 mg/kg (SA25) or estradiol ((ESTR) 0.2 mg/kg) was administered orally to rats, once daily for 28 days. SHAM: sham-operated control rats; OVX: ovariectomized control rats. Results are presented as the mean ± SEM. One-way ANOVA was used for evaluation of statistical significance of the results. No statistically significant differences in results for AST, ALT, uric acid, urea, and creatinine were demonstrated in ANOVA.

## Data Availability

Data will be made available upon request.
